# Identification of a Novel Canonical Splice Site Variant *TSC2* c.2967-1G>T That is Not Associated With Tuberous Sclerosis Pathogenesis

**DOI:** 10.3389/fgene.2022.904224

**Published:** 2022-05-27

**Authors:** Jing Duan, Yuanzhen Ye, Zhanqi Hu, Xia Zhao, Jianxiang Liao, Li Chen

**Affiliations:** Department of Neurology, Shenzhen Children’s Hospital, Shenzhen, China

**Keywords:** TSC2, alternative splicing, tuberous sclerosis, c.2967-1G>T, splicing variant, NAGNAG acceptor

## Abstract

Tuberous sclerosis, also known as tuberous sclerosis complex (TSC), is an autosomal dominant defect characterized by hamartomas in multiple organ systems. Inactivating variants cause this defect in either the *TSC1* gene or the *TSC2* gene, leading to hamartin or tuberin protein dysfunction, thus resulting in TSC. The diagnostic criteria for TSC suggest that it can be diagnosed by identifying a heterozygous pathogenic variant of *TSC1* or *TSC2*, even in the absence of clinical signs. In a 4-year-old girl, we identified a splicing variant (NM_000548.4: c.2967-1G>T) that she inherited from her father. Neither the girl (patient) nor her father showed typical features of TSC. This variant is located in a NAGNAG acceptor, which can produce mRNA isoforms that differ by a three-nucleotide indel. Reverse transcription polymerase chain reaction analysis of the patient and both parents’ blood RNA samples suggested two different splicing patterns, and these two splicing patterns differed in the presence or absence of the first codon of exon 27, thus providing two splicing products designated as isoforms A and B, respectively. Furthermore, the proportions of these two patterns varied between the patient and either parent. A minigene assay further confirmed that the c.2967-1G>T variant led to the absence of isoform A (including the first codon of exon 27). The finding of our study demonstrates this variant, c.2967-1G>T, disrupts the balance of an alternative splice event which involves the use of two tandem alternatives acceptors and is not associated with typical symptoms of tuberous sclerosis. Our finding is of importance for genetic counseling and suggests that we need to be vigilant to avoid misdiagnosis when we encounter such a site.

## Introduction

Tuberous sclerosis or tuberous sclerosis complex (TSC) is a neurocutaneous disorder that affects almost 2 million people worldwide and is characterized by uncontrolled benign tumor growth in multiple organ systems, such as the heart, lung, brain, and kidney (Northrup et al., 1993; Curatolo et al., 2015a; Curatolo et al., 2015b). TSC is associated with a high sporadic mutation rate, resulting from inactivating variants in *TSC1* (OMIM: 191100) and *TSC2* (OMIM: 613254) tumor suppressor genes (Curatolo et al., 2015b; Salussolia et al., 2019). The *TSC2* gene is located on chromosome 16p13.3 and was identified as a disease-causing gene of TSC in 1993 (European Chromosome 16 Tuberous Sclerosis C, 1993). *TSC2* encodes the 200-kDa protein tuberin, and inactivating variants in this gene usually cause a more severe clinical phenotype (Nellist et al., 2015). In most individuals with a confirmed diagnosis of TSC according to the Consensus Conference Clinical Diagnostic Criteria (Northrup et al., 2021), an inactivating variant in either *TSC1* (location: chromosome 9q34) or *TSC2* (location: chromosome 16p13.3) is considered diagnostic (Northrup et al., 1993). Furthermore, 11 major and 7 minor features comprise the clinical diagnostic criteria (Northrup et al., 1993; Curatolo et al., 2015b; Northrup et al., 2021).

The molecular diagnosis of TSC is established by identifying a pathogenic variant of *TSC1* or *TSC2*, irrespective of the clinical finding (Krueger and Northrup, 2013; Northrup et al., 2021). Since the clinical manifestation of TSC develops over time and at various ages, both interfamilial and intrafamilial variability are exhibited in TSC (Northrup et al., 1993; Krueger and Northrup, 2013). With the development of next-generation sequencing, there are several methods and strategies for clinical diagnosis, including identifying specific gene panels, whole-exome sequencing (WES), whole-genome sequencing, and multiplex ligation-dependent probe amplification. In addition, an increasing number of variants which may be associated with TSC pathogenesis are included in both *TSC1* and *TSC2* databases [e.g., Leiden Open Variation Database (LOVD)].

Among >10000 patients with TSC in whom pathogenic variants have been identified, a pathogenic variant of *TSC2* has been identified in ∼74% of patients, and a pathogenic variant of *TSC1* in the remaining patients (Northrup et al., 1993). Most of the reported pathogenic variants in the *TSC2* gene are truncating variants, including frameshift, nonsense, and splicing variants (Dabora et al., 2001; Au et al., 2007; Peron et al., 2018; Salussolia et al., 2019). A variant of the canonical +/-1 or 2 splice sites is often presumed to have a loss-of-function effect, particularly when the affected exon is present in a biologically relevant transcript (Abou Tayoun et al., 2018). Regarding *TSC2* variations, LOVD currently includes 238 single nucleotide variations of canonical +/-1 or 2 splice sites, and these variations are classified as either *likely pathogenic* or *pathogenic*. The early diagnosis of TSC facilitates genetic counseling, therapeutic intervention, and disease monitoring (Northrup and Krueger, 2013; Northrup et al., 2021). However, the wide variation in the TSC phenotype makes it challenging to establish a definitive clinical diagnosis of TSC, particularly in young patients. Despite the remarkable progress that has been made in TSC research, conventional molecular tests fail to identify a pathogenic *TSC1* or *TSC2* variant in 10–15% of TSC patients. These patients are usually referred to as TSC patients with no pathogenic variant identified (Qin et al., 2010; Tyburczy et al., 2015).

Herein, we identified a *TSC2* variant of canonical -1 splice site (c.2967-1G>T) in a patient without any features of typical TSC. We performed *in vitro* and *in vivo* assays to show that this variant leads to alternative usage of a NAGNAG tandem acceptor and affects the proportion of different transcripts. Our results suggest that c.2967-1G>T is not associated with typical symptoms of TSC; thus, in the future, special attention to similar splicing variant is needed in TSC patients.

## Materials and Methods

### Sample Collection

The Ethics Committee of the Shenzhen Children’s hospital approved this study. Peripheral venous blood samples were taken from a 4-year-old girl and her parents after obtaining informed consent. RNA was extracted using the TRIzol RNA reagent (TaKaRa, Shiga, Japan). In addition, DNA was extracted from the peripheral blood using the QIAamp DNA Mini Kit for blood (Qiagen, Hilden, Germany).

### Exome Sequencing

Trio-based WES was performed by KingMed Diagnostics (Guangzhou, China), including exome library preparation, sequencing, and data analysis. Libraries were generated using IDT’s xGen Exome Research Panel V1.0 (Coralville, IA, United States). The Illumina NovaSeq 6000 (Illumina, San Diego, CA, United States) with PE 150 was used to perform subsequent sequencing, with depth over 110x. Reads were aligned to the hg19 version of the human genome.

### Chromosomal Microarray Analysis

Chromosomal microarray analysis was performed using Affymetrix Cytoscan HD (Affymetrix, Santa Clara, CA, United States). The labeling and hybridization procedures were performed according to the manufacturer’s instructions. Herein, Chromosome Analysis Suite 4.2 (Affymetrix) was used to analyze raw data.

### *In Vivo* Assay

cDNA was synthesized from total RNA using the HifairTM 1st Strand cDNA Synthesis SuperMix (YEASEN, Shanghai, China). Then, the fragment spanning the exon 24–29 of *TSC2* was amplified using the following primers: 5′-CGG AAG GAT TTT GTC CCT TT-3′ and 5′- GCC TCC TTG GTC TGT CTC AC-3′. Finally, the polymerase chain reaction (PCR) product was verified by Sanger sequencing.

### Minigene Assay

The Minigene assay was performed by Bioeagle Biotech Co., Ltd. (Wuhan, China). Two pairs of nested primers—7198-*TSC2*-F (5′-CCG​CAC​CTC​TAC​AGG​AAC​TT-3′) and 11287-*TSC2*-R (5′-GAC​TGA​GCC​GAG​CTG​AAA​AC-3′) as well as 7491-*TSC2*-F (5′-TAG​CCA​TGT​GGT​TCA​TCA​GG-3′) and 10918-*TSC2*-R (5′-GAAGAGAATCCCACGCACAG-3′)—were designed to amplify the genomic region spanning the exon 25–27 of *TSC2* from the genomic DNA. The PCR-amplified fragments were cloned into the minigene vector pcDNA3.1. The recombinant vectors were transfected into 239T and MCF-7 cells. After 48 h, total RNA was isolated using the TRIzol reagent (TaKaRa). In the final stage, RT-PCR and Sanger sequencing were performed to analyze recombinant vector splicing.

### RNA Sequencing

To investigate the proportion of different transcripts in controls, we analyzed the already available RNA sequencing (RNA-seq) data of four randomly selected unrelated individuals: one healthy female adult, one healthy male adult, and two male children with neurological disorders other than TSC. They all had RNA-seq data available (the children for diagnoses and the adults for another research project). Their blood was collected in PAXgene Blood RNA tubes, and RNA was extracted using PAXgene Blood RNA Kit (PreAnalytiX, Hombrechtikon, Switzerland). RNA samples were subjected to RNA-seq *via* Aegicare (Shenzhen, China) using KAPA mRNA HyperPrep Kit (New England Biolabs, MA). The Illumina NovaSeq 6000 with PE150 (Illumina, San Diego, CA, United States) was used to perform subsequent sequencing. The same bioinformatic pipeline as previously described was used to analyze data (Maddirevula et al., 2020; Wai et al., 2020). Reads were aligned to the human genome (GRCh38).

## Results

### Identification of the c.2967-1G>T Variant

A 4-year-old girl was admitted to our hospital for epilepsy and developmental delay. She was born to healthy nonconsanguineous Chinese parents. There was no remarkable history of neurological disorders in this family, except for her father’s uncle being deaf and mute. She was born at full term following an uneventful pregnancy, but the birth weight was only 2000 g. Her developmental milestones were delayed. She could not roll over in both directions until the age of 8 months and could not sit without support until 11 months. Motor regression was observed at the age of 18 months without obvious predisposing causes, and she could not sit without support until 2.5 years of age, at which time she was diagnosed with epilepsy. She was given levetiracetam and valproic acid, which can reduce seizure frequency from 5 to 8 times a day to 2 times a day.

Trio-based WES was performed, revealing a splicing variant NM_000548.4:c.2967-1G>T in *TSC2*. This variant is not present in population databases (1000 Genomes, Exome Aggregation Consortium, and Genome Aggregation Database). The loss-of-function variants in *TSC2* are pathogenic (Northrup and Krueger, 2013). Additionally, this variant is also classified as *likely pathogenic* in the ClinVar database (National Center for Biotechnology Information, 2019). Interestingly, based on sequencing data and upon verification by Sanger sequencing, this variant was confirmed to have been inherited from her father, who had no history of seizures or other neurological problems despite having this variant. He is a healthy man who works for the government in China; he undergoes regular annual medical check-ups. The most recent check-up (including cardiac and abdominal ultrasound) was six months before this article was written, and it showed no abnormalities. Neither the patient nor her father presented with typical skin symptoms of TSC, such as hypomelanotic macules, angiofibromas (≥3), fibrous cephalic plaque, fibrous cephalic plaque, and Shagreen patch. The father did not undergo magnetic resonance imaging (MRI) of the head. Head magnetic resonance imaging and computed tomography scan of the patient performed at the age of 3 years was normal. Furthermore, cardiac and abdominal ultrasound of the patient performed at the age of 3 years was normal as well. Taken together, there were no clinical or radiographic findings supporting a diagnosis of TSC in the patient or her father. For the abovementioned reasons, the patient and her father were not considered as having TSC based on their clinical symptoms; however, the genetic results prompted a pediatric neurologist to conduct a clinical evaluation of the patient and his father.

### *In Vivo* Analysis of Splice Site Variant (c.2967-1G>T)

**FIGURE 1:**
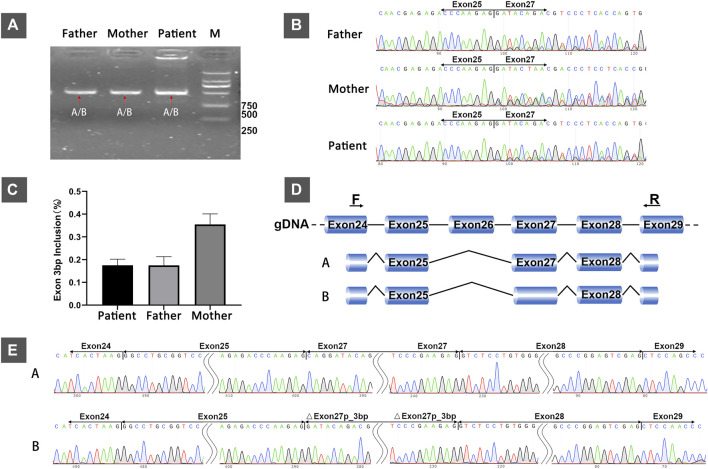
*In vivo* analysis of splicing: **(A)** Agarose gel electrophoresis of RT-PCR products from the 4-year-old female patient with developmental delay and her healthy parents. **(B)** Sanger sequencing of RT-PCR products from the patient and her parents showing double peaks, indicating two different splicing patterns. **(C)** The relative proportions of isoform A (with the first codon of exon 27) in the patient and her parents. **(D)** The schematic representation of the two different splicing patterns (isoforms A and B). **(E)** The sanger sequencing results of subcloning of the PCR product showing that the difference between the two transcripts was based on the absence or presence of the first codon of exon 27.

RT-PCR analysis of RNA extracted from the patient and her parents revealed that they all had only one PCR band (Figure 1A). However, Sanger sequencing of PCR products showed double peaks (Figure 1B), indicating two different splicing products (designated isoforms A and B) in all three samples. The proportion of two splicing patterns varied between the patient and her father or mother (without variant) (Figure 1C). Subcloning of the PCR product revealed that isoforms A and B both lacked exon 26. Conversely, the two isoforms differed in that the first codon in exon 27 was absent in isoform B. Therefore, these data confirmed that isoform A was [exon 24 (30 bp)–exon 25 (95 bp)–exon 27 (165 bp)–exon 28 (153 bp)–exon 29(39 bp)] and isoform B was [exon 24 (30 bp)–exon 25 (95 bp)–△exon 27 (162 bp)–exon 28 (153 bp)–exon 29 (39 bp)] (reference transcripts: NM_000548.5) (Figures 1D,E).

### *In Vitro* Analysis of c.2967-1G>T on Splicing of *TSC2*

**FIGURE 2:**
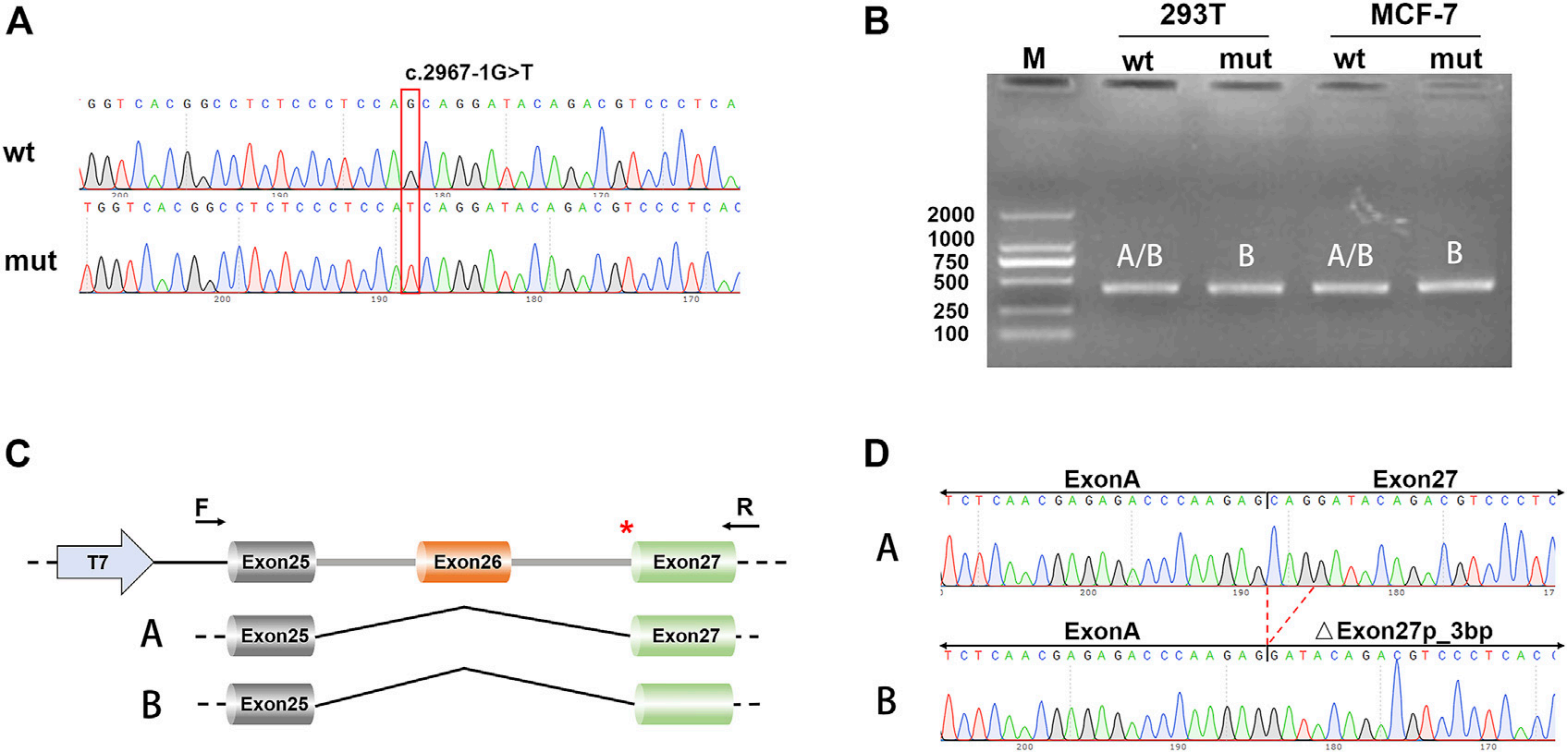
Minigene splicing assays. **(A)** Sanger sequencing results of the recombinant vector; the upper is WT and the lower is mut. **(B)** Electrophoresis results of transcript PCR products in both 293T and MCF-7 cell lines. **(C)** A schematic of cloned vectors and alternative splicing. **(D)** Sanger sequencing of the PCR products showing that the difference between the two transcripts was based on the absence or presence of exon 27.

We performed the minigene splicing assay to further reveal whether c.2967-1G>T influenced the splicing of *TSC2* in the genomic region spanning from exon 25 to exon 27 in *TSC2* (Figure 2A). We obtained two different minigenes by subcloning of exons 25–27 in the pcDNA3.1 vector: pcDNA3.1-*TSC2*–WT and pcDNA3.1-*TSC2*-mut. These two minigenes were transfected into 239T and MCF-7 cells, and RT-PCR was used to analyze the resultant transcripts. Subsequently, agarose gel electrophoresis of the PCR products showed a single PCR band (Figure 2B). However, Sanger sequencing of PCR products from the cDNA of pcDNA3.1-*TSC2*–WT showed double peaks, which represent two small transcripts. Subcloning of the PCR products from the cDNA of the pcDNA3.1-*TSC2*–WT minigene also revealed two different splicing patterns, namely A [exon 25 (95 bp)–exon 27 (165 bp)] and B [exon 25 (95 bp)–△exon 27 (162 bp)], corresponding to isoforms A and B during the *in vivo* analysis. However, Sanger sequencing of the PCR products from the pcDNA3.1-*TSC2*-mut minigene revealed only one splicing pattern [isoform B, exon 25 (95 bp)–△exon 27 (162 bp)] (Figures 2C,D), whereas the other splicing pattern corresponding to isoform A was absent [exon 25 (95 bp)–exon 27 (165 bp)].

### RNA-Seq Analysis of the Proportion of Different Transcripts in People Who do Not Have TSC

**FIGURE 3:**
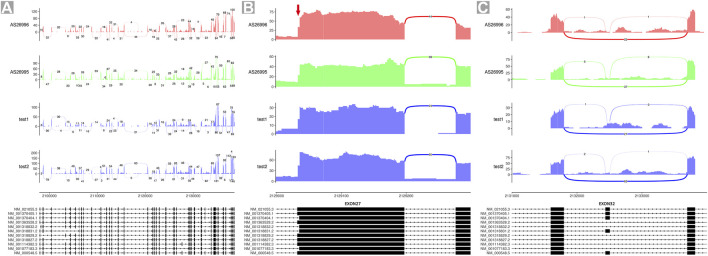
Sashimi plots of RNA-seq data of four individuals. **(A)** Quantitative visualization of alternative isoform expression in *TSC2* from RNA-seq data of four unrelated individuals. **(B)** Quantitative visualization of alternative isoform expression in exon 27 of *TSC2*. **(C)** Quantitative visualization of alternative isoform expression in exon 32 of *TSC2*.

Eleven different *TSC2*-coding transcripts are listed in the Gene database of the National Center for Biotechnology Information (NCBI; https://www.ncbi.nlm.nih.gov/gene/7249). To validate the proportion of isoforms A and B in controls, we analyzed the RNA sequencing data of blood samples of the aforementioned four unrelated individuals, including one healthy female adult (test 1), one healthy male adult (test 2), and two male children with neurological disorders other than TSC (AS26996 and AS26995). The data plotted as Sashimi plots suggest that all read maps crossed between exon 25 and exon 27, indicating the absence of exon 26 in the four samples (Figure 3A). Furthermore, the first codon in exon 27 was absent in about 63–66% of reads (isoform B) while the other reads map to this codon (isoform B) (Figure 3B). Few reads in these four samples were mapped to exon 32, indicating that transcripts encoding exon 32 are relatively sparse (Figure 3C).

## Discussion

The two tumor suppressor genes *TSC1* and *TSC2* are associated with the onset of TSC. *TSC2* variants account for approximately 70% of all disease-causing variants (Salussolia et al., 2019). *TSC2* encodes tuberin and is widely expressed in several organs and cell types (Northrup et al., 1993). Eleven different coding transcripts are listed in NCBI’s Gene database. Among these transcripts, NM_000548.5 is the longest transcript and the MANE select transcript recommended by the NCBI and the European Molecular Biology Laboratories-European Bioinformatics Institute. In our study, nucleotide and exon numbering also corresponded to NM_000548.5.

We report a splicing variant c.2967-1G>T in *TSC2,* which was identified by clinical genetic testing. This variant is located in canonical -1 splice site, and variations in this site are often presumed to have a loss-of-function effect. Interestingly, this splice variant could not be found in 1000 Genomes, Exome Aggregation Consortium, and Genome Aggregation Database, suggesting that it is a novel variant. Therefore, c.2967-1G>T was classified as *pathogenic*. However, this variant was inherited from the patient’s father, who had no history of seizures or other neurological problems. Moreover, the patient did not have clinical symptoms or radiographic tests indicative of a diagnosis of TSC either. Thus, we believe that this variant may not be a cause of TSC. In addition, we found that this variant was located in a NAGNAG receptor, which has been recorded in the TAndem Splice Site DataBase (TassDB, http://www.tassdb.info) (Sinha et al., 2010). The NAGNAG acceptor includes two 3′ tandem acceptors and mainly results in the insertion/deletion of one amino acid (Hiller et al., 2006). These findings outline the need for deep awareness of this variant and its implications in TSC. Therefore, the *in vivo* assay and minigene splicing assay were performed to reveal the impact of c.2967-1G > T.

Interestingly, our *in vitro* and *in vivo* studies could not detect exon 26 in blood samples. This finding may be attributed to alternate splicing, as similar results were reported by Ekong *et al.* (Ekong et al., 2016). Ekong *et al.* (Ekong et al., 2016) reported that exon 26 was undetectable in the blood and various tissues, including some tissues associated with TSC symptoms, such as the brain, kidney, lung, and liver. Additionally, in agreement with previous studies, our RNA-seq data of the four individuals also showed that exon 32 was detectable only in blood samples. Therefore, these data support that alternative splicing of exon 26 and exon 32 is common in the *TSC2* gene.

Our RT-PCR results of the mother who did not have this variant revealed two different splicing patterns, representing isoform A [exon 25 (95 bp)–exon 27 (165 bp)] and isoform B [exon 25 (95 bp)–△exon 27 (162 bp)] (the first codon in exon 27 was absent). This result confirms the effect of the NAGNAG acceptor and is consistent with previously reported effects of NAGNAG. Furthermore, the first codon in exon 27 is reportedly absent in some transcripts of *TSC2* in rodents and humans (Xiao et al., 1995; Xu et al., 1995). Most of the reported pathogenic variants in the *TSC2* gene were truncating variants (Northrup et al., 1993; Au et al., 2007). In particular, large genomic deletions (intragenic and whole-gene deletions) accounted for 16% of all variants reported (Cheadle et al., 2000). In addition, the haploinsufficiency score of *TSC2* in the ClinVar database is 3, which is sufficient evidence for haploinsufficiency. The triplosensitivity score of *TSC2* in the ClinVar database is 0, which means no evidence for triplosensitivity. Therefore, loss-of-function is the recognized mechanism of the *TSC2* variant leading to TSC. In addition, variants located in the canonical ±1 or 2 splice sites are often presumed to have loss-of-function effects because they often disrupt the open reading frame or exon skipping. However, the *in vivo* assay revealed that the patient and her father still had the two splicing patterns like the mother but the proportions of these two different isoforms differed. The minigene splicing assay showed that the WT construct yielded two different splicing patterns, which was in agreement with the *in vivo* assay results, whereas the mutant construct yielded a splicing pattern wherein the first codon of exon 27 was absent. We evaluated the loss of this amino acid in the context of two different protein sequences (NP_000539.2 and NP_066399.2) using the PROVEAN protein tool (Choi et al., 2012). The difference between the two protein sequences was in terms of whether or not they contained the amino acid encoded by exon 26. The PROVEAN scores for the absence of this amino acid were 0.516 (NP_000539.2) and −1.337 (NP_066399.2). The prediction (cut-off = −2.5) was neutral. Our results suggest that the c.2967-1G > T variant disrupts the balance of an alternative splicing event (formerly called alternative splice to NAGNAG acceptors), which involves the use of two alternative acceptors only 3 bp apart. Alternative splicing at NAGNAG acceptors is widespread and contributes to proteome plasticity (Hiller et al., 2004), and such NAGNAG acceptor sites can ameliorate or bypass the phenotype of a variant (Hinzpeter et al., 2010). This may explain the absence of typical symptoms of TSC in the patient and her father in our study.

According to our results, the pathogenicity of the c.2967-1G>T variant is uncertain. To identify the genetic causes leading to the illness of the patient, we reanalyzed the trio-WES data of our patient. A compound heterozygous variant in *TUBGCP6* [NM_0204461: c.4507G>A (p.Ala1503Thr) paternal and c.2492C>T (p.Ser831Phe) maternal] was found in the patient. These two variants were classified as variants of uncertain significance according to the American College of Medical Genetics and Genomics guidelines (Richards et al., 2015). Our patient’s symptoms only partially fit the spectrum of a *TUBGCP6*-related disease. In addition, to our knowledge, no functional connection between *TSC2* and TUBGCP6 has been reported so far. Therefore, the genetic cause of this patient’s clinical diagnosis is still unclear.

In conclusion, we identified a novel canonical -1 splice site variant, c.2967-1G>T, in *TSC2* in a patient without clinical symptoms of TSC. Our results indicate that the c.2967-1G>T variant disrupts the balance of an alternative splice event which involves the use of two alternatives acceptors. The findings of our study demonstrate that the variant c.2967-1G>T is not associated with typical symptoms of TSC. One of the important limitations of our study is that we cannot rule out whether the absence of isoform A due to this variant is without any effect on the function of the TSC protein complex; furthermore, we cannot rule out the presence of a relationship between the variant and epilepsy in the patient. Another limitation of our study lies in its availability of resources. Though we found the variant in NAGNAG acceptor affected splicing pattern and thereby the relationship of the variant with phenotype, it was studied in only one family. Therefore, future studies with a larger population cohort are needed to confirm the effect of similar variants in NAGNAG acceptors.

## Data Availability

The datasets presented in this study can be found in online repositories. The name of the repository and accession number can be found below: ClinVar, NCBI; SCV002499644.

## Abbreviations

ACMG, American College of Medical Genetics; TSC, Tuberous sclerosis.
